# Household decision dynamics and food insecurity: evidence from the *one-cow-per-poor-family* programme in Rwanda

**DOI:** 10.1007/s43546-025-00904-w

**Published:** 2025-08-28

**Authors:** Olayinka Idowu Kareem, Mayokun Akeremale, Christine Wieck, Theogene Dusingizimana, Olivier Kamana, Mizeck G. G. Chagunda

**Affiliations:** 1 Chair of Agricultural and Food Policy, University of Hohenheim, 70599 Stuttgart, Germany; 2 Department of Animal Breeding and Husbandry in the Tropics and Subtropics, University of Hohenheim, 70599 Stuttgart, Germany; 3 Department of Food Science and Technology, College of Agriculture, Animal Sciences and Veterinary Medicine, University of Rwanda, P.O. Box 210, Musanze, Rwanda; 4 Department of Applied Research and Development and Foresight Incubation, National Industrial Research and Development Agency, P. O. Box 273, Kigali, Rwanda

**Keywords:** Food insecurity, Household food insecurity, Decision dynamics, Livestock, Ordered probit, Rwanda

## Abstract

Rwanda, like many African countries, endeavours to overcome the food insecurity challenge with different initiatives. One such initiative is the ‘*one-cow-per-poor-family’* programme known as the ‘Girinka Programme’. With the introduction of every cow into the household, there is a resultant change in household bio-economics. Subsequently, some decisions must be made regarding either the consumption or the selling of milk, calves or manure. This study investigates the effect of household decision dynamics within the framework of this programme on food insecurity in Rwanda. The micro-econometrics analytical approach is adopted in the empirical strategy using the household data from the ‘Girinka Programme’ in an ordered probit model. A total of 3000 households were surveyed, and data were collected across 20 districts in Rwanda in 2016 and 2017 and used in the empirical estimations. Our findings affirm the a priori expectation that households’ food insecurity is reduced with additional income sources. Besides, the results indicate that the households acquired and/or used more land by participating in the programme through access to cow’s manure and income earned either by selling the heifer or milk, than before the programme. Also, we find that households with a higher number of calves are more food-secure. The estimations indicate that there is heterogeneity in the level of food insecurity across the districts owing to the decision dynamics of the households across the districts, which lead to different outcomes. The study concludes that participation in the Girinka Programme's intervention significantly reduced food insecurity. To this end, there is a need to establish, as a strategic policy option, the institutionalisation and strengthening of the Girinka Programme for sustainability, which should be affirmed and safeguarded as a social protection policy that directly impacts food insecurity and supports the attainment of sustainable development.

**Supplementary Information:**

The online version contains supplementary material available at 10.1007/s43546-025-00904-w.

## Introduction

Despite divergent national strategies, achieving food security is a common objective across countries. This aspiration becomes a challenge, particularly in Africa, because of endogenous factors such as political mismanagement, porous operationalisation, weak institutions and exogenous factors such as climatic conditions, land arability, plague, pandemics, location and demographic dynamics that impact the food system and consequently how any household has access to food. Experiencing food insecurity is a vital measure of poverty and a characteristic of underdevelopment. Household food insecurity (HFI) is the inability of all households to physically and economically either access or afford sufficient safe and nutritious food, both in quantity and quality, at all times, which satisfies the dietary need for healthy living (FAO, 2009; Habyarimana 2015; Agho et al. 2019; Rutayisire et al. 2023). The incidences of food insecurity occur whenever food security is limited or uncertain (Owino et al. 2014) Moreover, unsustainable land use, climate change and environmental degradation have also impacted the resilience of the food system, which affected the socioeconomic activities in the food value chains, thereby aggravating hunger, malnourishment and food insecurity, especially among the vulnerable groups in developing countries (Bedasa and Deksisa 2024; Maniriho 2024).

Rwanda, like many other developing countries, is faced with the challenge of food insecurity among its teeming population, especially among women and children, which was extensively aggravated during the civil war between 1990 and 1994. Emerging evidence has shown a prevalence of inadequate dietary diversity in Rwandan rural households, in which more than 50% of households have no direct access to sufficient food (Ansoms et al. 2017). This impacted heavily on household members’ welfare, particularly women and children, as 45% of children below the age of five are stunted and 12.6% of them are underweig^2024^ht, while anaemia is prevalent in women of reproductive age (Agho et al. 2019). Moreover, the contribution of the agricultural sector to the gross domestic product has continuously decreased from 44% in 1996 to 27% in 2023 (World Bank, ). Besides, the population growth^1^ has contributed to a serious level of hunger (Global Hunger Index 2024) which was impacted by the prevalence of undernourishment, severe and moderate food insecurity and employment in agriculture in total employment, which reduced from 90% in 1996 to 56% in 2022 (World Bank, 2024). The increasing population, agricultural employment reduction and low agricultural technology adoption have consequences on food insecurity. Population growth increases food demand, which exacerbates food insecurity (Habyarimana 2015; Iyakaremye and Kabanda 2024; Swain and Nsabimana 2024) while inadequate agricultural technology adoption affects the extent to which countries can feed their population.

Thus, engagement in agricultural activities is perceived as a reliable solution to tackling the issue of food insecurity at the household level since 70% of rural Rwandan households rely on agriculture for their livelihood strategy (Agho et al. 2019; Swain and Nsabimana 2024). To this end, increasing food production through sustainable agricultural practices is a tool for tackling the issues of food insecurity (Misselhorn et al. 2012). The Rwandan government has implemented heterogeneous policies and programmes that could enhance resilience to food insecurity and stimulate the transformation of the food system to cater for the rising population, poverty, severe food insecurity during the lean season, the prevalence of undernourishment and the mitigation against climate change. Among the government programmes to reduce food insecurity are the Strategic Plan for the Transformation of Agriculture (SPTA) in 2004, the Crop Intensification Programme (CIP) in 2006, the National Strategy for Transformation (NSTI), the Vision 2020 Umurenge Programme (VUP) in 2008, and the one-cow-per-poor-family called ‘Girinka programme’ launched in 2006. Nevertheless, there are also private food security intervention initiatives such as the United States-based nongovernmental organisations’ Food Security and Livelihood Programme (FSLP) launched in 2013 by the Partners in Health and Livelihood Donation Programme, the Plant Clinic Programme by the Plantwise Programme in 2011. These policies and programmes focus on different segments of Rwandan society and have heterogeneous outcomes. This study distils the effects of the Girinka programme (particularly the dynamic decisions made), which is a notable food insecurity resilience livestock programme among poor households, on rural households. For some time, livestock husbandry was considered an important pathway to take households out of poverty and food insecurity (Maniriho 2024; Danso-Abbeam et al. 2024; Chagunda et al. 2018; Nsabuwera et al. 2015; Rawlins et al. 2014).

The Economic Development and Poverty Reduction Strategy (EDPRS) in Rwanda describes livestock production as an important subsector within the agricultural sector and a strategic pillar in the achievement of a food and nutrition-secure society (Mazimpaka et al. 2017; Bizimana et al. 2012). To overcome the challenges of food insecurity, the Rwandan government initiated, as part of the EDPRS, the *one-cow-per-poor-family* ‘Girinka Programme’ that enabled each selected poor household to receive a cow. With this, it aims to increase agricultural productivity, thereby improving the income and livelihood strategies of rural households. Within the programme, households had the decision and/or choice to either sell or consume the milk or sell the calves. The first heifer calf then had to be passed on to another poor family. Manure from the animals would be used as fertiliser for crop production. Thus, this programme was designed to reduce food insecurity among the households since many of the households derived their livelihood from agriculture. The outcome of the households’ decision-making is at the centre of interest in this study, which informed the research question: to what extent have the household decision dynamics impacted food insecurity in the *one-cow-per-poor-family* ‘Girinka Programme’ in Rwanda?

Thus, the objective of the current study is to investigate the effect of household decision dynamics on food insecurity in the ‘Girinka Programme’ in Rwanda. The decision dynamics investigated consisted of the following choices: whether to consume or sell the produced milk and the heifers. Household food insecurity is operationalised in three different ways (further explained below), and the physical and capacity dimension is captured by socioeconomic and household characteristics. The findings indicate that the household decision to either consume or sell the produced milk and heifer has significantly reduced food insecurity among the programme-participating households.

A review of the literature indicates that different empirical strategies have been used to investigate the issues of food (in)security, especially in the context of Africa (Agho et al. 2019; Alinovi et al. 2018; Babatunde and Qaim 2009; Habyarimana 2015; Swain and Nsabimana 2024; Ngarava 2022; Tabe-Ojong and Nshakira-Rukundo 2023; Bedasa and Deksisa 2024; Danso-Abbeam et al. 2024). Suweis et al. (2015), Sisha (2020), Ratcliffe and McKernan (2010), Alinovi et al. (2018), and Iyakaremye and Kabanda (2024) have applied the mixed method; however, studies such as Ansah et al. (2019), Tendall et al. (2015) used the qualitative empirical approach to investigate the challenges of food insecurity, especially in rural areas. Beyond this, some studies have systematically reviewed the food (in)security literature to distil the food security impact of the different households’ decisions and also the approaches that were used to arrive at the outputs (Ansah et al. 2019; Bizikova et al. 2020; Tabe-Ojong and Nshakira-Rukundo 2023; Mumin and Abdulai 2022; Bedasa and Deksisa 2024). Moreover, some of the empirical studies in this area investigate the impact of household decision dynamics on food security (Owino et al. 2014; Ndakaza et al. 2017; Babatunde and Qaim 2009; Ratcliffe and McKernan 2010). The household decision dynamics arise because of different options available to the household to mitigate any possibility of food insecurity and/or shocks to food security that will impact the household’s welfare. Many of the empirical studies on the household decision dynamics response to food security were often characterised by binary/logistic structural modelling (logit, probit, Tobit, multilevel logit – and categorical models) and multinomial logit, multinomial probit, conditional logit, etc. (Sisha 2020; Suweis et al. 2015; Owino et al. 2014; Ndakaza et al. 2017; Habyarimana 2015; Danso-Abbeam et al. 2024; Mataka et al. 2023; Iyakeremye and Kabanda 2024). Many of the available studies on Rwanda, such as Iyakaremye and Kabanda (2024), Rutayisire et al. (2023), Ndakaza et al. (2017), and Habyarimana (2015), used binary or categorical outcomes models to investigate households’ food insecurity in different regions of Rwanda by classifying households into those that were food secure and food insecure, which were measures of the outcome variable. However, Danso-Abbeam et al. (2021) and Maniriho (2024), who applied the ordered probit model in the investigation of issues that affect food insecurity in Rwanda, did not explore, evaluate and/or determine the impact of the one-cow-per-poor-family national food security intervention programme on food insecurity as is done in this study.

Thus, this study contributes to the frontier of knowledge in this area of food security, especially in the context of Africa, as the empirical analysis of the targeted vulnerable household food insecurity-resilience programmes is yet to be analysed using a panel of homogenous households’ survey data measuring household food insecurity access scale (Rawlins et al., 2014; Chagunda et al. 2018; Nsabuwera et al. 2015; Rutayisire et al. 2023; Swain and Nsabimana 2024). Besides, the distillation of the food security impact of the one-cow-per-poor family (Girinka Programme) in Rwanda is yet to be undertaken or explored in the literature to ascertain the effectiveness and the extent of food insecurity resiliency of the Girinka programme (Jean de Dieu and Vital 2020; Chagunda et al. 2018; Swain and Nsabimana 2024). More so, this study empirically disaggregates the food security impact of the Girinka programme across 20 districts covering all the provinces. Hence, the contributions of this study are established in the context of extant studies such as Chagunda et al. (2018), Jean de Dieu and Vital (2020), Nsabuwera et al. (2015), Maniriho (2024), Nsabimana et al. (2020), Danso-Abbeam et al. (2021), Iyakaremye and Kabanda (2024), Danso-Abbeam et al. (2024), Swain and Nsabimana (2024) that investigate the trends and potential factors that characterised household food security using the micro and macro data such as the Rwandan Comprehensive Food Security and Vulnerability Analysis Survey 2012 to 2018, cross-sectional populated-based survey conducted every biennial from the National Institute of Statistics of Rwanda, Rwandan integrated household living condition surveys 2013/14 and 2016/17. These were generic studies that investigated the factors that influence, determine and/or impact household food security; they did not explore the specific impact of any food security intervention programmes. However, food insecurity-specific intervention programmes such as Rawlins et al. (2014) investigate the impact of the heifer international donation programme and Nsabuwera et al. (2015) assess the effects of an integrated food security intervention in three Rwandan districts, neither of these studies explores the analysis of the effects of the national food security intervention programme such as Girinka Programme on food insecurity as being investigated in this study. Besides, Habyarimana (2015) and Danso-Abbbeam et al. (2021) evaluate the determinants of food insecurity in rural households in Rwanda; however, they did not extend the analysis to examine the impact of the programme implemented on food security as investigated in this study. The vulnerable household food insecurity programme-specific study by Chagunda et al. (2018) investigates the genetic diversity and population structure of dairy cattle in smallholder dairy systems using the high-density single-nucleotide polymorphism in the Girinka programme; however, the study did not focus on the household food security aspect of the programme as investigated in this study. It is on the basis of the identified gaps that this study is situated.

Apart from this introductory section, the following section deals with the study’s methodology, which includes the model and the description of the study areas. The third section gives the empirical results, while the fourth section provides an extensive discussion of the results, and the last section concludes.

## Methods

This study’s empirical strategy employs a micro-econometrics method—a quantitative analytical approach. The method makes use of data from the field surveys of rural Rwandan households to analyse the food security situation among the households in the programme. Also, uses the survey data to estimate ordered probit models that allow the analysis of the link between household livestock (cows) decision-making and food security at the household and regional levels.

### Study region

**Fig. 1:**
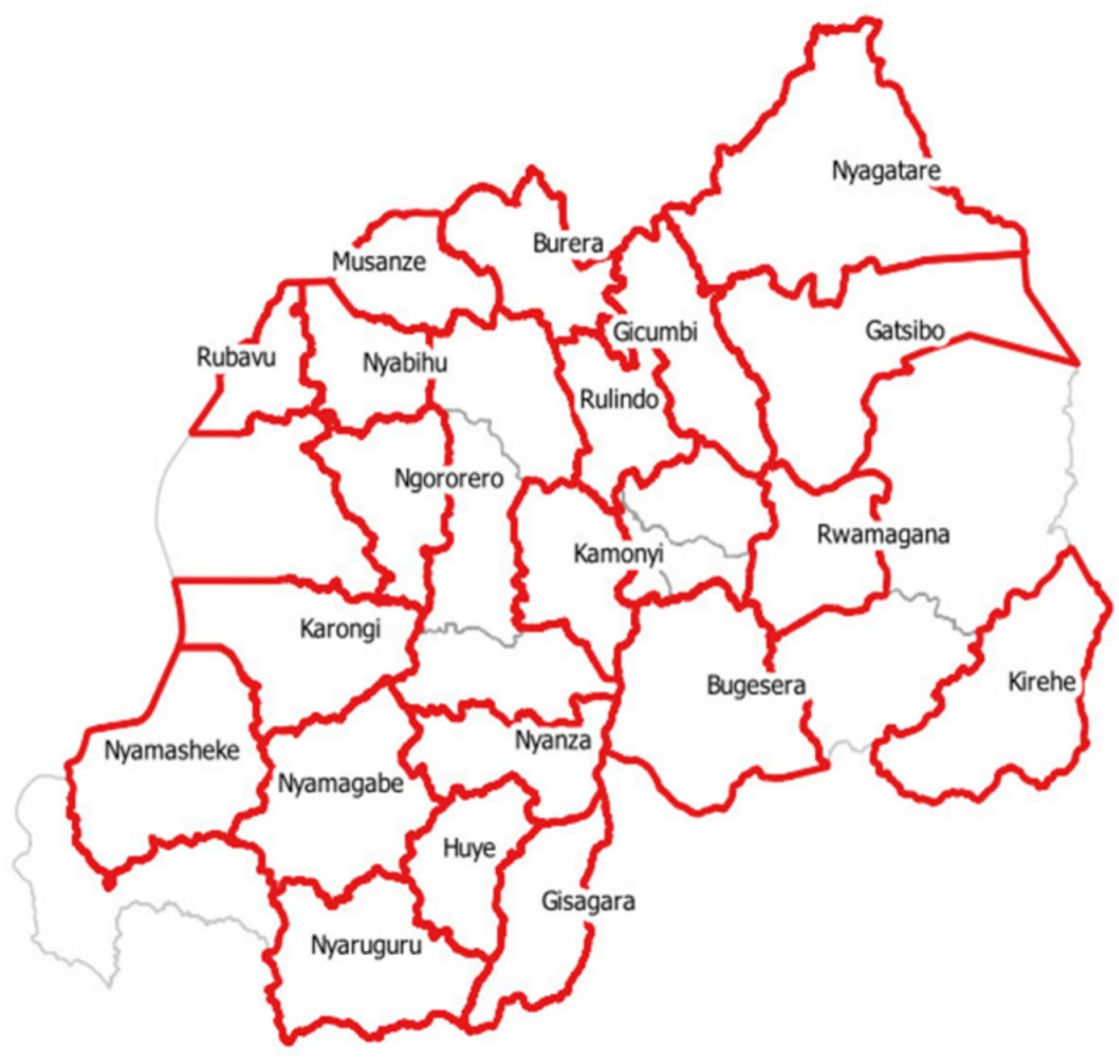
Map of Rwanda showing the districts of focus

This study covers twenty districts in the Northern, Southern, Eastern and Western regions of Rwanda (see appendix Table 2). Out of the 3000 households covered in the field survey, six hundred and twenty-three interviewed households were in the Northern province, nine hundred and forty-one households interviewed were in the Southern province, seven hundred and seventy-two were in the Eastern province, and six hundred and sixty-four were in the Western province of Rwanda. The districts covered in the survey are shown in Fig. 1.

### Field survey

The empirical analysis was based on primary data collected using a survey of 3000 households. The broad aim of the survey was to evaluate the potential of the *one-cow-per-poor-family* initiative as a source of animal germplasm for dairy cow development. Specifically, it examines how the cow received within the programme translates into current food security and future dairy breeding. Since the survey was conducted via face-to-face interviews with household members, it is possible to ascertain the impact of the programme on food security at the household and district levels.

The sampling frame for the Girinka Programme was collected from the programme office in Rwanda. A purposive random sampling technique was adopted to select the sample size, which involves a purposive selection of districts across all regions of the country where the dairy farmers in the programme were located. The sampling technique was adopted because only the recipients of cows in the programme fit the objective of this study and were targeted for sampling. Within the districts, the interviewed households were randomly selected.^2^ The purposive sampling technique is a non-probability sampling method that allows the selection of targeted respondents who will provide the most appropriate and useful information (Ahmed 2024; Kelly 2010). In the selection of the sample size adjustment was made for the size/population and the regional distribution of the programme’s recipients. A digitalised, structured questionnaire implemented through the Open Data Kit (ODK) was used to interview selected households (see Chagunda et al. 2018 for details). The survey was conducted in 2016 and 2017.^3^ An account of the dependent variables’ questions, related variables, and summary statistics can be found in Appendix Table 1.

A section of the administered questionnaire consists of the nine Household Food Insecurity Access Scale (HFIAS) questions that collate households’ behaviour and responses to food insecurity thirty days before the survey. These questions were universal guidelines used in estimating the frequency of household food insecurity (Hamilton et al. 1997). They have been based on the awareness that the occurrence of food insecurity prompts predictable reactions that, if monitored through a survey, could be recorded and quantified into a scale (Coates et al. 2007a, b). The scale, then, is used to distinguish between food-secure and food-insecure households (Hamilton et al. 1997). This same measurement of household food insecurity has been proven to be an effective and efficient global measurement used in capturing information on household food access status. The derived information then serves as a tool in the monitoring and assessment of geographical targeting of the prevalence of food insecurity (Hamilton et al. 1997; Webb et al. 2006; Carletto et al. 2013). Questions about resources management such as the decision on milk management – consumption and sales, milk income, number of calves, heifers, choice of cow, commercial cow feed purchase, money spent on water for the cow and the difficulty faced in selling the milk were included to understand food insecurity triggered by resources management decisions. Socioeconomic factors have been proven to influence food security at the household level (Carletto et al. 2013). Thus, the households’ socio-economic indicators that were considered are household head age, gender, education, marital status, household size, and household land size – before and after the project – and source of income. It is acknowledged that the seasonality of the field survey might impact pastures for the cows, which could affect food availability and household decision-making.

### Quantitative analysis

#### Model description

There have been theoretical frameworks underpinning the implementation of empirical strategies in this area of research (Owino et al. 2014; Suweis et al. 2015; Ansah et al. 2019; Sarma 2024). For instance, the item response theory, which postulates that the probability of a household’s certain reaction to a stimulus is a function characterising the household food insecurity level on a latent trait, was used as the framework to derive the Rasch model for the analysis of food insecurity (Owino et al. 2014). The Engel curve was adopted by Nsabimana et al. (2020) within the theoretical framework of consumer behaviour to investigate household food demand, in which unconditional consumption elasticities of food were computed for urban and rural households in Rwanda. Sarma (2024) combined the random utility and decision-making threshold theories to determine eligibility for livestock and dairy development and the response that only occurs when the stimulus’s intensity surpasses each person’s threshold for reaction. Iyakaremye and Kabanda (2024) determine the impact of family income and size on food security using a logistic model within the framework of ecological-evolutionary theory that describes the relationship among agricultural practice, policy, economic disparity and food security.

The theoretical framework for the empirical model in this study is adopted from Matchaya and Chilonda (2012), which builds on Sadoulet and de Janvry (1995) food demand frameworks by incorporating the consumer utility optimisation framework and the household characteristics that determine preferences and/or decisions. The food demand framework (Sadoulet and de Janvry 1995) integrates production, labour and consumption factors and/or decisions simultaneously as determinants. However, the Matchaya and Chilonda (2012) framework further assumes that the factors that determine food demand also influence household food security. Hence, food demand factors were integrated into the modelling of household food security. The household food security reduced form equation was specified as:

1 $$Y_{i}^{*} = \beta_{0} + \sum\nolimits_{i = 0}^{k} {\beta_{j} } X_{ij} + \varepsilon_{i}$$

where ${Y}_{i}^{*}$ is the household food availability, a measure of food security; ${X}_{ij}$ is the vector of explanatory variables for the household $i,$ which encompasses household characteristics and the socioeconomic variables that influence household decisions as a producer, consumer and income earners, ${\beta }_{0}$ is the intercept, ${\beta }_{j}$ are the coefficients while ${\varepsilon }_{i}$ stands for the error term in which the nature of the dependent variable determines its distributional structure. The household food need (demand) is linked to food security in the sense that if household food demand falls below the minimum, then there is household food insecurity. Thus, the factors that determine food security (${Y}_{i}^{*}$) also impact food demand. Furthermore, Sadoulet and de Janvry (1995) and Matchaya and Chilonda (2012) took the dependent variable (${Y}_{i}^{*}$) as a continuous variable and hence, analysed the model using the ordinary least squares regression estimator.

However, this study modifies the Matchaya and Chilonda (2012) framework to integrate the intra-household economic decision dynamics in the ‘Girinka Programme – *one-cow per-poor-family initiative*’ in Rwanda, which includes milk management, milk income, male and female calves, as well as the heifers. The modification is the form of the extension of the factors that determined food insecurity to three categories; food availability, accessibility and stability This is because, beyond household worry that there would not be enough food, it is pertinent to determine whether the household is constraint by lack of resources to consume the preferred quantity of food it wanted. These are linked to ${Y}_{i}$ because if the household does not have enough or access to food, then it is food insecure. Hence, based on the conceptualisation of food security in the context of the programme investigated in this study, our food security function is specified as follows:

2 $${Y}_{i}=f\left({IHDD}_{i}, {IHIF}_{i}, {X}_{i}\right), i=1,\cdots ,n.$$

The a priori expectation marginal effects of Eq. 2 (the partial derivatives) are:

3 $$\frac{{dY_{i} }}{{d1HDD_{i} }} > 0,\frac{{dY_{i} }}{{d1HIF_{i} }} > < 0,\frac{{dY_{i} }}{{dX_{i} }} > < 0.$$

Given these partial derivatives, the reduced form equation of the food security function is specified as:

4 $${Y}_{i}^{*}={\beta }_{0}+{\beta }_{j}\sum_{i=0}^{k}({IHDD}_{i}+{IHIF}_{i}+{X}_{ij})+{\varepsilon }_{i}$$

where ${Y}_{i}^{*}$ is the household food security, ${IHDD}_{i}$ is the intra-household decision dynamics,${IHIF}_{i}$ represents the intra-household influencing factors, ${X}_{ij}$ is the vector of household characteristics that influence household decisions as a producer, consumer and income earner, ${\beta }_{0}$ is the intercept, ${\beta }_{j}$ are the variable coefficients, while ${\varepsilon }_{i}$ stands for the error term as stated in Eq. 1. The intra-household decision dynamics,${IHDD}_{i},$ of the ‘Girinka Programme’ is measured by the *one-cow per-poor-family initiative* milk management, milk income, male calves, female calves and the heifers. The ${IHIF}_{i}$ is measured by household socioeconomic factors such as landholding size, credit accessibility, off-farm income, and training received. Other household characteristics,${X}_{ij}$, is the age of the household head, the gender of the household head, education, etc.

Household food security is measured in three ways. Although nine different variables are used in the ‘Girinka Programme’ in Rwanda to ascertain whether the household is food-secured or not, they are categorised into three in this empirical modelling – household food availability, food accessibility and food stability. The three categories are used as measures of food security in the models as dependent variables. Another modification to the Matchaya and Chilonda (2012) framework is the adoption of the ordered probit model within the framework of McKelvey and Zavoina (1975) with a system of equations. This ordered probit model assumes a nonlinear effect of each explanatory variable and a series of breakpoints between the categories of the dependent variable. The choice of this model is due to the ordinal response and categorical nature of the outcome variable, which the binary models fail to account for. Besides, the system of ordered probit regression equations allows the specification of different outcome equations, whereby each equation represents the structural model for the ‘Girinka programme’ food insecurity measures. In addition, in the circumstances where the responses are ordinal, it is the ordered probit model that is often adopted.

McKelvey and Zavoina (1975) built the ordered probit model based on an unobserved random variable called a latent variable that is generally specified as follows (Nkegbe et al. 2019):

5 $$Y_{i}^{*} = X_{i}^{\prime } \beta + \varepsilon_{i} ,\,\,\,\,\,\,i = 1,2, \ldots ,N$$

where ${Y}_{i}^{*}$ is the unobserved (latent) random dependent variable; $X_{i}^{\prime }$represents the vector of the regressors; $\beta$ is the vector of the parameters (coefficients) to be estimated, while ${\varepsilon }_{i}$ stands for the error term that takes the characteristics of $E\left({\varepsilon }_{i}|{X}_{i}\right)$ = 0 and $Var\left({\varepsilon }_{i}|{X}_{i}\right)=1$. Since the theoretical ${Y}_{i}^{*}$ cannot be directly observed, a latent variable; it is proxied by an observable variable ${Y}_{i}$, which are the ordinal and categorical variables with j responses. The vector of the unobservable threshold parameters is defined as:

6 $$\alpha = {\alpha }_{-1}, {\alpha }_{0}, {\alpha }_{1}, \dots , {\alpha }_{j-1}, {\alpha }_{j}$$

Therefore, the interaction between the unobserved (latent) dependent variable and the observed dependent variable is stated as follows:

7 $$If\alpha_{j - 1} < Y_{i}^{*} \le \alpha_{j} ,{\text{then}}Y_{i} = j.\,\,\,\,\,\,\,\,\,\,\,j = 1,2,3, \ldots ,k.$$

Besides, ${\alpha }_{-1}= -\infty , {\alpha }_{0}=0, {\alpha }_{j}=\infty ,$ while it is also the case that ${\alpha }_{-1}<{\alpha }_{0}<{\alpha }_{1}\dots <{\alpha }_{j}$.

The probability of the occurrence of the observed dependent variable is specified as:

8 $$\begin{aligned} Prob\left[ {Y_{i} = j} \right] = & Prob\left[ {\alpha_{j - 1} < Y_{i}^{*} \le \alpha_{j} } \right] \\ & = Prob\left[ {\alpha_{j - 1} - X_{i}^{\prime } \beta < \varepsilon_{t} \le \alpha_{j} - X_{i}^{\prime } \beta } \right] \\ & = \phi \left[ {\alpha_{j} - X_{i}^{\prime } \beta } \right] - \phi \left[ {\alpha_{j - 1} - X_{i}^{\prime } } \right] \\ \end{aligned}$$

where $$\Phi \left(.\right)$$ represents the standard cumulative distribution function, while j is the dependent variable response classification. As stated earlier, the latent variable ($${Y}_{i}^{*})$$ is unobservable, which gives the basis for an observable proxy variable ($${Y}_{i})$$ that is specified as:

$${Y}_{i}\left\{\begin{array}{c}0 if{Y}_{i}^{*}\le 0 \\ 1 if 0<{Y}_{i}^{*}\le {\alpha }_{1}\\ 2 if {\alpha }_{1}<{Y}_{i}^{*}\le {\alpha }_{2}\end{array}\right.$$ (9)

Extending Eq. 9 to the observable dependent variable in the ‘*one-cow per-poor-family initiative’* of the Girinka Programme in Rwanda, we specify outcome variable responses as:

$${Y}_{i}\left\{\begin{array}{c}1 if{Y}_{i}^{*}\le 0 \\ 2 if 0<{Y}_{i}^{*}\le {\alpha }_{1}\\ 3if {\alpha }_{1}<{Y}_{i}^{*}\le {\alpha }_{2}\end{array}\right. \equiv {Y}_{i}\left\{\begin{array}{c}1 if rarely food insecure \\ 2 if sometimes food insecure\\ 3 if often food insecure\end{array}\right.$$ (10)

Thus, Eq. 10 is the ordered probit model for ordinal responses of the dependent variable. To internalise the ordered probit model in this study, we re-specify Eq. 4 in line with the ordinal responses in Eq. 10 as a system of equations.^4^ The specification of the system of equations is based on the fact that food insecurity in the household is measured from three perspectives – household food availability, food accessibility and food stability.

$${Y}_{1}={\beta }_{0}+{\beta }_{j}\sum_{i=0}^{k}({IHDD}_{i}+{IHIF}_{i}+{X}_{ij})+{\varepsilon }_{i}$$ (11)

$${Y}_{2}={\beta }_{0}+{\beta }_{j}\sum_{i=0}^{k}({IHDD}_{i}+{IHIF}_{i}+{Y}_{1}+{X}_{ij})+{\varepsilon }_{i}$$ (12)

$${Y}_{3}={\beta }_{0}+{\beta }_{j}\sum_{i=0}^{k}({IHDD}_{i}+{IHIF}_{i}+{Y}_{1}+{Y}_{2}+{X}_{ij})+{\varepsilon }_{i}$$ (13)

The variables’ definition is as given in Eq. 4; besides, $${Y}_{1}$$ is the household food availability, $${Y}_{2}$$ represents household food accessibility and $${Y}_{3}$$ stands for household food stability. In addition to the a priori expectation in Eq. 4, food availability ($${Y}_{1})$$, measured by food production, is estimated independently of food accessibility ($${Y}_{2})$$ and food stability ($${Y}_{3})$$, while $${Y}_{2}$$ is estimated independently of $${Y}_{1}$$ and $${Y}_{3}$$, and $${Y}_{3}$$ estimation is independent of $${Y}_{2} and {Y}_{3}$$.

#### Data description

The variables used in the empirical strategy are derived from the ‘Girinka Programme's field survey of twenty districts in Rwanda in 2016 and 2017. In the survey instrument,^5^ nine of the questions deal with food insecurity, which is the measure of the HFIAS. The nine questions are the nine variables in the survey dataset used for the HFIAS, which were disaggregated into three categories – food availability, food accessibility and food stability, where each category comprises three variables (questions). Each of the categories is measured by one of the three variables in the empirical estimations.^6^ Food availability is measured by these three variables: households worry about enough food, households eat smaller portions than required, and households eat less than needed. The food accessibility variables are: due to inadequate resources, the households are unable to eat the preferred food; households eat limited food varieties due to inadequate resources; and households eat unwanted food due to inadequate resources. While the households ever had no food of any kind to eat, households slept at night hungry, and households went the whole day and night without food are used to measure food stability (see Table 1 in the appendix for the data descriptive statistics and definitions). In the baseline estimation (Table 6), a combination of households worrying about enough food, households eating limited food varieties due to inadequate resources and households having no food of any kind to eat are used for food availability, accessibility and stability, respectively. The average of these disaggregated food insecurity variables (average of food availability, accessibility and stability measurements) is used for the aggregated food insecurity variable in the baseline estimation. The disaggregated districts’ estimation also combines the variables: households eat fewer than needed, households eat unwanted food due to inadequate resources, and households go the whole day and night without food, while the food insecurity estimation uses the average of these combined variables. For the robustness check, another set of variables is combined. For instance, food availability is measured by households eating smaller portions than required, households unable to eat the preferred food and households sleeping at night hungry measure food accessibility and stability, respectively. The average of these disaggregated food insecurity variables defined the aggregated food insecurity in the robustness check.

The variables measuring HFIAS were coded as 1 if they rarely occur (once or twice in the past four weeks), 2 if they sometimes occur (three to ten times in the past four weeks), and 3 if they often occur (more than ten times in the past four weeks). The households’ characteristics and the socio-economic variables enter the estimations in different indices. For instance, the gender of households’ heads is coded 1 if it is male, while a female is 2. The choice of the cow received is coded 1 for yes and 2 for no responses, respectively. The households’ decisions on heifers are whether they were sold, 1; passed on to other programme participants, 2; kept, 3; or others, 4.

## Results

### Field survey analysis

Out of the 3000 households (HH) surveyed for this study, 55% were male-headed and 45% female-headed (Table 1) .

**Table 1:** Overview of the land size distribution by gender of the household head. Source: Own calculation based on the survey

	Total	Total HH Land size (ha)			
Gender of HH Head		< 0.5	0.5–1	1.1–2	2.1–3
Female	1,342	32,2%	10,9%	1,5%	0,1%
Male	1,658	40,6%	12,2%	2,3%	0,1%
Total	**3,000**	**72,8%**	**23,1%**	**3,8%**	**0,3%**

^a^See appendix II for the sample size by districts

Regarding locations, 623 interviewed households were in the Northern districts, 941 households interviewed were in the Southern districts, 772 were in the Eastern districts, and 664 were in the Western districts of Rwanda. Turning to the development of the land size holding of each household, we compare land size before joining the ‘Girinka Programme’ and after receiving a cow. As Table 2 shows, before joining the programme, about 87% of households displayed a land size holding of < 0.5 ha and only 2% had an average land size of 1-3 ha. The remaining households worked on holdings between 0.5-1 ha. Except for the largest size class, the number of households led by male household heads was larger. After receiving a cow, the percentage of households working on holdings of very small land size (< 0.5 ha) decreased, indicating that about 4% of the participating households were able to increase their holdings in terms of land size. Increases were observed across the other three class sizes. Both male and female household heads were about equally able to increase their land size. This invariably implies that households were able to expand their agricultural activities, specifically food production, after receiving the cows in the programme, which improves the households’ food security.

**Table 2:** Comparison of household land size holdings before and after joining the programme. Source: Own calculation based on the survey

Gender of HH head	Before receiving a cow				After receiving a cow			
	Land size of holding (in ha)				Land size of holding (in ha)			
	< 0.5	0.5–1	1.1–2	2.1–3	< 0.5	0.5–1	1.1–2	2.1–3
Female	38,6%	5,4%	0,6%	0,1%	36,5%	7,5%	0,7%	0,1%
Male	48,1%	6,0%	1,0%	0,1%	45,9%	7,6%	1,6%	0,1%
Total	86,7%	11,4%	1,7%	0,2%	82,4%	15,1%	2,2%	0,3%

Next, the focus is on sources of income and major income earners in the household. The majority of the interviewed households (50%) relied on other sources of income and the sale of crops (26%) for a stream of income, as stated in Table 3. The income stream from the sale of livestock products only accounted for 4% of the total interviewed households. Within households, about 51% responded that there was no single household income earner, while 47% stated the household heads as the major household income earners. No obvious differences could be observed when focusing on the gender dimension.

**Table 3:** The household’s major income earner. Source: Own calculation based on the survey

	Source of Income						Major Income earner			
Gender of HH Head	Full-time paid employment	Part-time paid employment	Own Business	Crop sales	Livestock	others	Household head	child	partner	No single earner
Female	1,2%	10,8%	3,5%	29,1%	4,8%	50,6%	46.0%	1.9%	0.6%	51.5%
Male	1,5%	16,4%	5,1%	24,2%	3,7%	49,1%	48.1%	0.9%	0.4%	50.7%
Total	1,4%	13,9%	4,4%	26,4%	4,2%	49,7%	47.2%	1.3%	0.5%	51%

Finally, in the survey, some questions regarding intra-household decision-making were asked. The findings show (Table 4) that the most agreed-upon household decision regarding the heifers was to keep them to expand the herd size (42%), followed by selling (30%). However, about 27% of the households also passed the (first) heifer of the cow on to another family to fulfil a requirement of the programme. The household dynamic decisions expanded food production and the outreach of the programme, and improved households' food insecurity because the expansion of a household’s herd size directly impacts food security.

**Table 4:** Households’ decisions on how to use the heifers (by region). Source: Own calculation based on the survey

	Decision on Heifer			
Region	Sell	Pass on	Keep	Other
West	30,2%	17,2%	52,6%	0%
East	37,2%	17,8%	44,9%	0%
South	19,4%	34,5%	44,3%	1,7%
North	34,8%	37,9%	25,0%	2,2%
Total	29,6%	27,1%	42,3%	1,0%

Regarding the key decision-maker on income generated from milk sales (Table 5), a considerable proportion of the households’ (46%) milk income decisions were jointly made by the male and female household heads. However, 36% of the households reported that “no decision” was taken, indicating that the household has yet to decide on the allocation of the milk income.

**Table 5:** Responsibility for households’ decisions on milk income (by region). Source: Own calculation based on the survey

	The decision on milk income				
Region	No decision	Myself	Man	Woman	Joint Decision
West	1%	7,3%	4,9%	2,1%	84,8%
East	0,6%	10,1%	5,4%	2,4%	81,5%
South	68,3%	13,9%	3,7%	3,2%	10,8%
North	69,2%	9,8%	4,8%	1,8%	14,4%
Total	36,2%	10,6%	4,6%	2,5%	46,1%

Thus, the main findings from the analysis of the field survey are that the landholdings of the households have increased due to this programme; half of the households relied on other sources of income, while the household decision on the heifer was built and/or expanded their herd size. Finally, most household decisions were made jointly.

### Estimation results

As aforementioned, ordered probit models are estimated using four different dependent variables. First, results are presented for the full sample (Table 6); second, a focus on differences across districts was made (Table 7).

**Table 6:** The estimations of the Girinka programme’s impacts by food security categorization**.** Source: Estimated

	Food Insecurity	Food Availability	Food Accessibility	Food Stability
Household head age	0.104 (0.076)	0.098^**^ (0.047)	0.157^***^ (0.042)	-0.375^***^ (0.093)
Household head gender	-0.707^**^ (0.325)	-0.124 (0.155)	-0.173 (0.126)	-0.476 (0.354)
Household head education	-0.224 (0.142)	-0.286^***^ (0.068)	0.000 (0.056)	-0.578^***^ (0.158)
Household head marital status	0.008 (0.083)	0.050 (0.047)	-0.025 (0.042)	0.163^*^ (0.096)
Household size	0.060^*^ (0.031)	0.074^***^ (0.016)	-0.001 (0.014)	0.111^***^ (0.031)
Source of Income	-0.224^***^ (0.057)	-0.096^***^ (0.028)	0.059^**^ (0.025)	-0.110^**^ (0.052)
Total income	0.000 (0.000)	-0.000^***^ (0.000)	-0.000^***^ (0.000)	-0.000^***^ (0.000)
Average land size	-0.155 (0.125)	0.590^***^ (0.142)	-0.272^***^ (0.083)	-0.986^***^ (0.356)
Land size before	0.534^*^ (0.278)	0.240^**^ (0.103)	0.487^***^ (0.087)	0.132 (0.250)
Land size after	-1.149^***^ (0.291)	-0.935^***^ (0.165)	-0.337^***^ (0.101)	0.984^**^ (0.437)
Cow choice	-0.705^***^ (0.231)	0.081 (0.118)	0.114 (0.087)	-1.286^***^ (0.219)
Calves number	-0.656^*^ (0.348)	-0.255 (0.167)	-0.355^***^ (0.119)	-0.484 (0.333)
Feed purchase	-0.072 (0.187)	-0.085 (0.091)	-0.018 (0.085)	-0.454^***^ (0.172)
Water expenditure	-0.008^***^ (0.002)	-0.007^***^ (0.001)	-0.002^***^ (0.001)	0.006^***^ (0.002)
Heifers’ decision	-0.064 (0.072)	0.009 (0.036)	-0.100^***^ (0.031)	-0.390^***^ (0.084)
Milk sold	-0.103^**^ (0.051)	-0.108^***^ (0.024)	-0.060^***^ (0.021)	-0.173^***^ (0.049)
Milk consumed	-0.149^**^ (0.061)	-0.059^*^ (0.030)	-0.042^*^ (0.023)	0.045 (0.067)
Milk sales difficulty	-0.382^***^ (0.122)	-0.122^*^ (0.066)	0.071 (0.059)	-0.565^***^ (0.145)
Edu*Calves number	0.230^***^ (0.073)	0.117^***^ (0.038)	0.026 (0.030)	0.315^***^ (0.066)
Gender*Calves number	0.267 (0.172)	0.066 (0.074)	0.178^***^ (0.054)	0.075 (0.179)
Observations	419.000	1560.000	1917.000	448.000
Wald Chi^2^	205.390 (0.000)	369.980 (0.000)	317.900 (0.000)	226.200 (0.000)

All the variables are estimated at their levels. The figures in parentheses are the robust standard errors of the estimates. The significant variables are denoted by the *, ** and *** at 10, 5 and 1 per cent significance levels, respectively.

**Table 7:** The disaggregated estimation of the programme’s food security impacts by districts. Source: Estimated

Districts^a^	Food Insecurity	Food Availability	Food Accessibility	Food Stability
Gicumbi	0.480^***^ (0.093)	0.459^***^ (0.060)	0.642^***^ (0.055)	−0.022 (0.095)
Huye	0.355^***^ (0.103)	0.840^***^ (0.062)	1.012^***^ (0.060)	−0.163 (0.105)
Gisagara	0.228^**^ (0.090)	0.216^***^ (0.060)	0.324^***^ (0.060)	−0.323^***^ (0.101)
Burera	−0.431^***^ (0.098)	0.108^*^ (0.058)	0.187^***^ (0.057)	−0.304^***^ (0.092)
Nyaruguru	0.855^***^ (0.115)	0.635^***^ (0.073)	1.171^***^ (0.061)	0.167 (0.104)
Kamonyi	1.048^***^ (0.084)	1.158^***^ (0.062)	1.077^***^ (0.058)	1.041^***^ (0.082)
Nyanza	0.997^***^ (0.084)	1.493^***^ (0.081)	1.257^***^ (0.061)	−0.240^**^ (0.118)
Rulindo	0.970^***^ (0.097)	0.874^***^ (0.066)	0.690^***^ (0.059)	0.127 (0.112)
Musanze	−1.032^***^ (0.118)	−0.618^***^ (0.114)	−0.701^***^ (0.089)	−1.804^***^ (0.212)
Gatsibo	0.830^**^ (0.342)	0.188^***^ (0.054)	−1.814^***^ (0.149)	0.132 (0.243)
Bugesera		0.345^***^ (0.058)	−5.684^***^ (0.041)	
Karongi	0.093 (0.143)	0.111^*^ (0.063)	−1.133^***^ (0.108)	−0.123 (0.204)
Nyamasheke	−0.474^*^ (0.277)	0.057 (0.063)	−1.291^***^ (0.108)	−0.103 (0.216)
Rwamagana		−0.280^***^ (0.063)	−5.684^***^ (0.041)	
Nyagatare	−5.775^***^ (0.128)	−0.059 (0.062)	−5.684^***^ (0.041)	−4.915^***^ (0.124)
Kirehe		−0.063 (0.058)	−5.684^***^ (0.041)	
Nyabihu		0.085 (0.062)	−5.684^***^ (0.042)	
Ngororero		−0.210^***^ (0.065)	−5.684^***^ (0.041)	
Rubavu		0.291^***^ (0.056)	−5.684^***^ (0.042)	
Observations	2334.000	12,583.000	11,099.000	2770.000
Wald Chi^2^	4100.930 (0.000)	1484.590 (0.000)	146,118.370 (0.000)	2859.050 (0.000)

The figures in parentheses are the robust standard errors of the estimates. Some districts dropped due to collinearity, and the similarity in districts’ responses contributed to the homogeneous coefficients. The significant variables are denoted by the *, ** and *** at 10, 5 and 1 per cent significance levels, respectively.

The salient facts of the estimates^7^ in Table 6 indicate that the gender of the household heads significantly determines food security, such that female-headed households experience a reduction in food insecurity by 70% compared to their male counterpart. Maniriho (2024) also found that the likelihood of a reduction in food insecurity in female-headed households with livestock farming, while Iyakaremye and Kabanda (2024), Jean de Dieu and Vital (2020) and Habyarimana (2015) found that male-headed households were more likely to achieve food security. The household size is significant to the issue of food insecurity, as households with larger sizes have the challenge of food insecurity, such that an additional person will increase household food insecurity by 6%. Sarma (2024) and Danso-Abbeam et al. (2024) findings also align with the fact that food insecurity is aggravated among large household sizes. This means that households with many members, especially in rural areas, often have difficulty attaining food security, which could further impoverish the households; however, Mataka et al. (2023) findings indicate food security improves with more household members. The results also show that the more the households’ income sources, the better for the households, as food insecurity will be significantly reduced by 22% for every additional source of income, which affirms the theoretical expectation that households’ food insecurity is alleviated with additional income sources. Moreover, the households’ total income significantly reduces food insecurity, though the magnitude and/or the extent to which it propels food security is indistinguishable from zero (0.00%) for every additional household’s income. Further, the estimates indicate that households’ land ownership (size) after participating in the Girinka Programme significantly impacts food insecurity; for instance, for every additional plot acquired before the programme, the food insecurity within the households tends to rise by about 15%. However, after participating in the programme, the land size tends to reduce food insecurity, such that for every additional land acquired, the food insecurity in the households plummets by more than 100%, which conforms with Maniriho (2024). This implies that the manure obtained from the cow distributed in this programme stimulates interest in expanding land size due to improved crop yields. Also, we find that having a choice of the cow to receive in the programme did not significantly reduce the households’ food insecurity. This implies that the households that were given cows without making a cow choice got their food insecurity reduced by 70% for every zero-decision made on the choice of cow received. Besides, the number of calves that the cow produced significantly reduced households’ food insecurity to the extent that food could be secured by 65% for every additional calf produced, which affirms Rawlins et al. (2014) and Maniriho (2024) findings.

In terms of the households’ decision on milk management, the results show that the households that decided to consume milk got about a 15% reduction in food insecurity, while the food insecurity for those households that sold the milk plummeted by 10%. These results imply that the households’ food insecurity is better reduced by the decision to consume the produced milk. This means that it might be the case that not all proceeds from the sales of milk would be used by the households to provide food. Besides, there might be a withdrawal of the amount made from milk sales for other purposes not related to overcoming the households’ food insecurity. More so, the more the households have access to the market to get their milk sold for better prices, the greater the reduction in food insecurity, to the extent that the households’ food insecurity is reduced by 38% for every additional ease of selling the milk. Jean de Dieu and Vital (2020) find that the possession of livestock is essentially suitable for overcoming food insecurity challenges. Moreover, the number of calves that were produced by the better-educated households’ heads (edu*calves number) presents the fact that food insecurity was not reduced when the calves' production decision was made by them. There was a tendency that their decision might have aggravated food insecurity by 23% for every decision made, which also implies that higher education attainment did not influence the number of calves produced in the ‘Girinka Programme’.

Summarising the findings up to this point, the results show that the households’ decisions on the choice of cow received, calves’ number and whether to sell or consume the milk significantly reduced the level of food insecurity. However, the decision on heifers is insignificant to the households’ food insecurity. Besides, while gender did matter in the number of calves produced to reduce the households’ food insecurity, the level of education did not. Hence, overall, the ‘Girinka programme’ reduces the beneficiary households’ food insecurity.

In the second step, four models are estimated across the districts to explain differences in the four food security variables. Given the estimation data, 20 districts are covered in the project survey and used in the estimation (see Appendix Table 2), but 6 districts were dropped due to the correlation. Besides, the first district – Nyamagabe – was taken as the baseline/reference point. The disaggregation of the estimations of the programme’s impact across the districts is shown in Table 7. The estimations indicate that there is heterogeneity in the level of food insecurity across the districts; while the programme had been able to alleviate the challenge of food insecurity in some districts, it was not the case for others. This is due to the decision dynamics of the households across the districts, which lead to heterogeneous outcomes.

Besides, the way this programme was implemented could also contribute to the disparity in food insecurity outcomes. For instance, out of the 19 districts estimated for food availability, only 5 districts – Musanze, Rwamagana, Nyagatare, Kirehe and Ngororero – were rarely worried and/or feared about the availability of food.^8^ The Musanze had the largest magnitude (0.62) of less anxiety and worries about the availability of food, while the lowest magnitude (0.05) went to Nyagatare, which means that Nyagatare tends towards being more worried about food availability and that the programme had not secured their food.

Overall, Nyanza was the most worried about whether the food would be enough – availability – with a significant impact of 1.49%, while the lowest food availability district is Nyamasheke, with an estimated significant impact of 0.05% for every cow received in the programme. For food accessibility, 11 out of the 19 estimated districts were able to access their preferred food. The districts of Bugesera, Rwamagana, Nyagatare, Kirehe, Nyabihu, Ngororero and Rubavu had the most significant access to varieties of food, such that the districts had on average 5.68% more access to food for every cow they received. However, Burera was the district with the most inability to access adequate food, such that for every cow received, there was 0.19% inadequate access to food. Furthermore, only 5 districts – Gisagara, Burera, Nyanza, Musanze and Nyagatare – had significant food stability throughout the project’s survey period, with direct impacts of 0.32%, 0.30%, 0.24%, 1.80%, and 4.92%, respectively, for every cow received. Thus, the aggregate food insecurity was reduced in 4 districts – Burera, Musanze, Nyamasheke, and Nyagatare – which could be among others, owing to the cows received **i**n the project. However, 8 districts – Gicumbi, Huye, Gisagara, Nyaruguru, Kamonyi, Nyanza, Rulindo and Gatsibo—experienced increased food insecurity.

### The robustness check

This section checks the robustness of the estimated results in Table 6 by re-estimating the ordered probit regression with a different set of dependent variables. That is, in this robustness check, we have selected another set of variables for the food security categories. The estimates in Table 8 for all the categories of food security indicate similarity with the estimates in Table 6, except that households’ income source becomes significant here. Besides, the intersections of education and calf number, as well as that of gender and calf number, are more significant in Table 6 than in Table 8. This shows that, irrespective of the HFIAS variables used to measure food insecurity, the estimates give similar outcomes.

**Table 8:** The Robustness check results. Source: Estimated

	Food Insecurity	Food Availability	Food Accessibility	Food Stability
Household head age	0.104 (0.076)	0.098^**^ (0.047)	0.157^***^ (0.042)	−0.375^***^ (0.093)
Household head gender	−0.707^**^ (0.325)	−0.124 (0.155)	−0.173 (0.126)	−0.476 (0.354)
Household head education	−0.224 (0.142)	−0.286^***^ (0.068)	0.000 (0.056)	−0.578^***^ (0.158)
Household head marital status	0.008 (0.083)	0.050 (0.047)	−0.025 (0.042)	0.163^*^ (0.096)
Household size	0.060^*^ (0.031)	0.074^***^ (0.016)	−0.001 (0.014)	0.111^***^ (0.031)
Source income	−0.224^***^ (0.057)	−0.096^***^ (0.028)	0.059^**^ (0.025)	−0.110^**^ (0.052)
Total income	0.000 (0.000)	−0.000^***^ (0.000)	−0.000^***^ (0.000)	−0.000^***^ (0.000)
Average land size	−0.155 (0.125)	0.590^***^ (0.142)	−0.272^***^ (0.083)	−0.986^***^ (0.356)
Land size before	0.534^*^ (0.278)	0.240^**^ (0.103)	0.487^***^ (0.087)	0.132 (0.250)
Land size after	−1.149^***^ (0.291)	−0.935^***^ (0.165)	−0.337^***^ (0.101)	0.984^**^ (0.437)
Cow choice	−0.705^***^ (0.231)	0.081 (0.118)	0.114 (0.087)	−1.286^***^ (0.219)
Calves number	−0.656^*^ (0.348)	−0.255 (0.167)	−0.355^***^ (0.119)	−0.484 (0.333)
Feed purchase	−0.072 (0.187)	−0.085 (0.091)	−0.018 (0.085)	−0.454^***^ (0.172)
Water expenditure	−0.008^***^ (0.002)	−0.007^***^ (0.001)	−0.002^***^ (0.001)	0.006^***^ (0.002)
Heifers decision	−0.064 (0.072)	0.009 (0.036)	−0.100^***^ (0.031)	−0.390^***^ (0.084)
Milk sold	−0.103^**^ (0.051)	−0.108^***^ (0.024)	−0.060^***^ (0.021)	−0.173^***^ (0.049)
Milk consumed	−0.149^**^ (0.061)	−0.059^*^ (0.030)	−0.042^*^ (0.023)	0.045 (0.067)
Milk sell difficulty	−0.382^***^ (0.122)	−0.122^*^ (0.066)	0.071 (0.059)	−0.565^***^ (0.145)
Edu*Calves number	0.230^***^ (0.073)	0.117^***^ (0.038)	0.026 (0.030)	0.315^***^ (0.066)
Gender*Calves number	0.267 (0.172)	0.066 (0.074)	0.178^***^ (0.054)	0.075 (0.179)
Observation	419.000	1560.000	1917.000	448.000
Wald Chi^2^	205.390 (0.000)	369.980 (0.000)	317.900 (0.000)	226.200 (0.000)

All the variables are estimated at their levels. The figures in parentheses are the robust standard errors of the estimates. The significant variables are denoted by the *, ** and *** at 10, 5 and 1 per cent significance levels, respectively.

## Discussion

The findings suggest that the gender of the household head directly impacts the food security situation, such that households headed by women significantly experience less food insecurity compared to the men-headed households in the programme. This could be because women are closer to the members of the household, especially to the children, and are often interested in ensuring that the household has enough food. This affirms the findings of Upton et al. (2016), Sisha (2020) and Alinovi et al. (2018). The size of the households is an important determinant of household food security, as the findings show that small households are more food secure than large households. Given the communal practices or extended family practices in rural areas, the household sizes are often large, especially for poor households, which contributes to the inability to be food secure. Our finding of a negative relationship between the size of households and food security aligns with findings by Ratcliffe and McKernan (2010) and Alinovi et al. (2018).

Regarding income sources, our findings are in line with the theoretical expectation that households’ food insecurity is alleviated with additional income sources and that the total income matters for the status of food security (Mustapha et al. 2016; Upton et al. 2016). In terms of the land size, the estimates confirm the field survey result that shows that the households acquired and/or used more land in the programme than before the programme. This presents the fact that the Girinka programme provides an avenue for more acquisition of land through the additional income derived from the sales of milk, heifers and manure. A similar result was obtained by Kumba et al. (2016) when they found that household food insecurity was reduced owing to the increase in agricultural land use. Focusing on the livestock, our finding that the number of calves has a direct impact on food security is confirmed by the result from Upton et al. (2016) that households with larger livestock holdings have more stable dietary diversity. Besides, Rawlins et al. (2014) and Danso-Abbeam et al. (2021) find household ownership of livestock as an important determinant of food security in Rwanda. This affirms the importance of vulnerable household intervention programmes to reduce food insecurity in Africa, particularly in Rwanda. This was confirmed by Lascano Galarza (2020) in Malawi in the food assistance programme called ‘The R4 Rural Resilience Initiative.’ Also, Walingo (2009) concludes that the livestock development projects in Kenya have the potential to improve households’ food and nutrient security. Danso-Abbeam et al. (2021) concurred about the importance of the one-coe-cow-per-household relevance in combating hunger and food insecurity in Rwanda.

## Conclusion

The increment in agricultural and food production or imports is a veritable channel through which countries, especially developing economies, can overcome the challenges of food insecurity. The Girinka Programme was initiated as a pro-poor programme by the Rwandan government to enhance households’ food production and livelihoods to reduce food insecurity and improve the standard of living in the country. An assessment of the programme is necessary to be able to ascertain its impact on households’ food security. It is on this basis that this study aims to determine the effects of the households’ decision dynamics in the programme – *one-cow-per-poor family* – on food insecurity. The mixed-method approach is adopted in the empirical strategy using the programme survey data in an ordered probit model. A total of 3000 households’ survey data that were collected in 2016 and 2017 are used in the empirical estimations.

This programme has been found to increase some households’ land-size holdings, while the households’ choice of the cows received is irrelevant to food insecurity. Besides, the calves produced in the programme were a means of overcoming households’ food insecurity. Further, the households’ decision to consume the produced cows’ milk significantly reduced food insecurity more than selling the cows’ milk, while improving market access and better prices for the cow milk reduced food insecurity. Thus, this programme has significantly contributed to the households’ food security in Rwanda. This study affirms the findings in the literature that enunciate the importance of households’ intervention programmes on food insecurity. The scaling up of this programme is encouraged in Rwanda, and a similar programme is recommended for other countries in Africa. Besides, the expansion of the coverage of the programme to include more poor households is necessary – as only about 13% of the households in the country are covered by the programme as of 2022 – given that the programme seems to have reduced the participating households’ food insecurities. Furthermore, a policy should be introduced that empowers the government to make direct choices of cows to the households because the cow choices made by the households significantly hamper their food security. There is a need for the Girinka Programme's milk consumption policy that ensures that a larger proportion of the milk produced by cows is consumed within the households to reduce food insecurity. Hence, the one-cow-per-poor-family programme should be institutionalised as a social protection policy for effectiveness and sustainability.

Although the field survey used in this study covers two-thirds of the districts in Rwanda, future studies could cover all the districts in the country. Moreover, given that the observations of this study’s empirical estimates did not exhaust the sample size in the survey owing to the surveyed households’ disparity in the two survey periods (2016 and 2017), we recommend that future panel field surveys of this programme should be done on the same households for higher observations for the empirical estimations and degree of freedom. Besides, since this study only covers the Girinka Programme, other national food security intervention programmes such as the Crop Intensification Programme and Vision 2020 Umurenge Programme could be investigated, while the nonstate initiatives such as livestock donation by Heifer International and Food Security and Livelihood Programme could be extensively investigated in future studies to ascertain their impact on food insecurity.

## Supplementary Information

Below is the link to the electronic supplementary material.

Supplementary file1 (DOCX 20 kb)

## Data Availability

The Girinka Programme’s one-cow-per-poor family field survey data is available at 10.6084/m9.figshare.7046768. https://figshare.com/articles/dataset/Use_of_high_density_single_nucleotide_polymorphism_SNP_arrays_to_assess_genetic_diversity_and_population_structure_of_dairy_cattle_in_smallholder_dairy_systems_the_case_of_Girinka_Programme_in_Rwanda/7046768

## Footnotes

1 Population more than doubled, from 5.7 million in 1995 to about 13.7 million in 2024 (NISR 2024).

2 The selected districts cut across all the regions of Rwanda.

3 These are the most recent survey data on this programme.

4 Models with observables outcomes.

5 Survey instrument and questionnaire are used interchangeably.

6 Three empirical estimations are carried out; baseline estimation (Table 6), disaggregated districts estimation (Table 7) and the robustness check (Table 8).

7 Note that the discussion focuses on the second column of Table 6, as the dependent variable food insecurity is direct and aggregated other dependent variables (see Table 1 in the summary material). In addition, we also estimated and displayed three alternative specifications on food availability, accessibility, and stability, that provide additional perspectives on food insecurity. Largely, the results for each explanatory variable across these four columns of dependent variables are the same. Hence, our focus on the dependent variable food insecurity,

8 See Table 7.
