# Supplementary material for: Household decision dynamics and food insecurity: evidence from the one-cow-per-poor-family programme in Rwanda
Source: SN Bus Econ. 2025 Aug 28;5(9):132. doi: 10.1007/s43546-025-00904-w (PMC12394342; doi:10.1007/s43546-025-00904-w)
Supplement: Supplementary file 1 — Supplementary file1 (DOCX 20 kb) [file 43546_2025_904_MOESM1_ESM.docx]

**APPENDIX**

# Table 1 Estimation variables’ definitions and descriptive statistics

| **Variable** | **Description** | **Obs.** | **Min** | **Max** |
| --- | --- | --- | --- | --- |
| Food insecurity is the average of the measures of food availability, accessibility and stability. | How often did you worry that your household would not have enough food? + How often did you or any household member have to eat a limited variety of foods due to a lack of resources? + How often was there ever no food to eat of any kind in your household?)/3. | 464 | 1 | 3 |
| Food insecurity_district estimation | How often did you or any other household member have to eat fewer meals in a day because there was not enough food? + How often did you or any household member have to eat some foods that you did not want to eat because of a lack of resources to obtain other types of food? + How often did you or any household member go a whole day and night without eating anything because there was not enough food? | 447 | 1 | 3 |
| Food insecurity_robustness check | How often did you or any household member have to eat a smaller meal than you felt you needed because there was not enough food? + How often were you or any household member not able to eat the kinds of foods you preferred because of a lack of resources? + How often did you or any household member go to sleep at night hungry because there was not enough food?)/3. | 430 | 1 | 3 |
| Food availability for the baseline estimation: households worry about having enough food. | In the past four weeks, how often did you worry that your household would not have enough food? Rarely – 1, sometimes – 2, often – 3. | 2,423 | 1 | 3 |
| Food availability for the district estimation: households eat smaller portions than required. | In the past four weeks, how often did you or any household member have to eat a smaller meal than you felt you needed because there was not enough food? Rarely – 1, sometimes – 2, often – 3. | 1,220 | 1 | 3 |
| Food availability for the robustness check: households eat fewer meals than needed. | In the past four weeks, how often did you or any other household member have to eat fewer meals in a day because there was not enough food? Rarely – 1, sometimes – 2, often – 3. | 1,022 | 1 | 3 |
| Food accessibility for the baseline estimation: households are unable to eat their preferred food due to a lack of resources. | In the past four weeks, how often were you or any household member not able to eat the kinds of foods you preferred because of a lack of resources? Rarely – 1, sometimes – 2, often – 3. | 2,594 | 1 | 3 |
| Food accessibility for the district estimation: households eat limited food varieties due to a lack of resources. | In the past four weeks, how often did you or any household member have to eat a limited variety of foods due to a lack of resources? Rarely – 1, sometimes – 2, often – 3. | 2,128 | 1 | 3 |
| Food accessibility for the robustness check: households eating unwanted due to a lack of resources. | In the past four weeks, how often did you or any household member have to eat some foods that you did not want to eat because of a lack of resources to obtain other types of food? Rarely – 1, sometimes – 2, often – 3. | 2,604 | 1 | 3 |
| Food stability for the baseline estimation: households ever had no food of any kind to eat. | In the past four weeks, how often was there ever no food to eat of any kind in your household because of the lack of resources to get food? Rarely – 1, sometimes – 2, often – 3. | 550 | 1 | 3 |
| Food stability for the district estimation: households sleep at night hungry. | 8a. In the past four weeks, how often did you or any household member go to sleep at night hungry because there was not enough food? Rarely – 1, sometimes – 2, often – 3. | 456 | 1 | 3 |
| Food stability for the robustness check: households went the whole day and night without food. | In the past four weeks, how often did you or any household member go a whole day and night without eating anything because there was not enough food? Rarely – 1, sometimes – 2, often – 3. | 281 | 1 | 3 |
| Household head age | The age of the head of households – a closed-ended question. | 3000 | 1 | 5 |
| Household head gender | The gender of the head of the household. Male – 1, female – 2. | 3000 | 1 | 2 |
| Household head education | The highest level of education of the head of household. | 3000 | 1 | 7 |
| Household head marital status | The marital status of the head of the household. | 3000 | 1 | 3 |
| Household size | The total number of household members – open-headed. | 3000 | 0 | 19 |
| Source of income | The major source of household income. | 2991 | 1 | 6 |
| Total income | The total income obtained from all the economic activities. | 2955 | 0 | 200000 |
| Average land size | The average land size – close-headed question. | 3000 | 1 | 4 |
| Land size before | Agricultural land size cultivated before the ‘Girinka Project’ – close-headed question. | 2999 | 1 | 4 |
| Land size after | Agricultural land size cultivated after implementing the ‘Girinka Project’ – closed-ended question. | 3000 | 1 | 4 |
| Cow choice | Did the households have a choice over the cow they received? Yes – 1, No – 2. | 3000 | 1 | 2 |
| Calves number | The number of calves from the cow is received. | 2230 | 0 | 39 |
| Feed purchase | Did the household ever purchase commercial feed for the cow? Yes – 1, No – 2. | 1585 | 1 | 2 |
| Water expenditure | The expenditure on water for the cow. | 2332 | 0 | 1000 |
| Heifers decision | The households’ decisions on heifers from the cow–sell (1), pass-on (2), keep (3), and others (4). | 2995 | 1 | 4 |
| Milk sold | Out of the milk produced, the amount that was sold is in litres. | 2241 | 0 | 28 |
| Milk consumed | Out of the milk produced, the amount that was consumed is in litres. | 2237 | 0 | 25 |
| Milk sells difficultly | Did the households ever have difficulties selling the milk? Yes – 1, No – 2. | 1810 | 1 | 2 |
| Edu*Calves number | The interaction of household heads' highest education attainment and the number of calves. | 2230 | 0 | 117 |
| Gender*Calves number | The interaction of household heads’ gender and the number of calves. | 2230 | 0 | 39 |
| Districts | The districts where the field survey was conducted were in Rwanda. | 3000 | 1 | 20 |

Source: Compiled and computed

# Table 2 Sample size by district

| **Districts** | **Sample survey** | **% of the total sample** |
| --- | --- | --- |
| Nyamagabe | 159 | 5.30 |
| Gicumbi | 263 | 8.77 |
| Huye | 154 | 5.13 |
| Gisagara | 173 | 5.77 |
| Burera | 153 | 5.10 |
| Nyaruguru | 198 | 6.60 |
| Kamonyi | 190 | 6.33 |
| Nyanza | 62 | 2.07 |
| Rulindo | 157 | 5.23 |
| Musanze | 55 | 1.83 |
| Gatsibo | 191 | 6.37 |
| Bugesera | 131 | 4.37 |
| Karongi | 120 | 4.00 |
| Nyamasheke | 155 | 5.17 |
| Rwamagana | 155 | 5.17 |
| Nyagatare | 158 | 5.27 |
| Kirehe | 137 | 4.57 |
| Nyabihu | 116 | 3.87 |
| Ngororero | 146 | 4.87 |
| Rubavu | 127 | 4.23 |

Source: Compiled and computed.
